# Community ambulation in older adults and people with OA – a model verification using Canadian Longitudinal Study on Aging (CLSA) data

**DOI:** 10.1186/s12877-023-04598-3

**Published:** 2024-01-06

**Authors:** Ruth Barclay, Yixiu Liu, Jacquie Ripat, Robert Tate, Scott Nowicki, Depeng Jiang, Sandra C. Webber

**Affiliations:** 1 Department of Physical Therapy, College of Rehabilitation Sciences, Rady Faculty of Health Sciences, University of Manitoba, R106-771 McDermot Ave, Winnipeg, Manitoba R3E 0T6 Canada; 2 Department of Community Health Sciences, Max Rady College of Medicine, Rady Faculty of Health Sciences, University of Manitoba, Winnipeg, Canada; 3 Department of Occupational Therapy, College of Rehabilitation Sciences, Rady Faculty of Health Sciences, University of Manitoba, Winnipeg, Canada

**Keywords:** CLSA, Community ambulation, Older adults, Osteoarthritis, Structural equation modeling

## Abstract

**Background:**

There are health and well-being benefits of community ambulation; however, many older adults do not regularly walk outside of their home. Objectives were to estimate the associations between latent constructs related to community ambulation in older adults aged 65–85 (65+), and in adults with osteoarthritis (OA) aged 45–85.

**Methods:**

Secondary data analysis of the comprehensive baseline and maintaining contact questionnaire data from the Canadian Longitudinal Study of Aging (CLSA) was completed. Based on a previous model of community ambulation post-stroke, structural equation modeling (SEM) was used to develop measurement and structural models for two groups: older adults 65+ and people with OA. Multi-group SEM was conducted to test measurement invariance across sex and age groups. Measurement models were developed for the following latent factors: ambulation (frequency of walking outside/week, hours walked/day, ability to walk without help, frequency and aids used in different settings); health perceptions (general health, pain frequency/intensity); timed functional mobility (gait speed, timed up-and-go, sit-to-stand, balance). Variables of depression, falls, age, sex, and fear of walking alone at night were covariates in the structural models.

**Results:**

Data were used from 11,619 individuals in the 65+ group (mean age 73 years ±6, 49% female) and 5546 individuals in the OA group (mean age 67 ± 10, 60% female). The final 65+ model had a close fit with RMSEA (90% CI) = 0.018 (0.017, 0.019), CFI = 0.91, SRMR = 0.09. For the OA group, RMSEA (90% CI) = 0.021 (0.020, 0.023), CFI = 0.92, SRMR = 0.07. Health perceptions and timed functional mobility had a positive association with ambulation. Depression was associated with ambulation through negative associations with health perceptions and timed functional mobility. Multi-group SEM results reveal the measurement model was retained for males and females in the 65+ group, for males and females and for age groups (65+, < 65) in the OA group.

**Conclusions:**

The community ambulation model post-stroke was verified with adults aged 65+ and for those with OA. The models of community ambulation can be used to frame and conceptualize community ambulation research and clinical interventions.

**Supplementary Information:**

The online version contains supplementary material available at 10.1186/s12877-023-04598-3.

## Background

Community ambulation is defined as “independent mobility outside the home, which includes the ability to confidently negotiate uneven terrain, private venues, shopping centers and other public venues” [1]. Community ambulation occurs when walking outside of one’s home and includes walking both indoors and outdoors (e.g., at a park, someone else’s home or at a shopping centre). Other related terms include community mobility and outdoor walking.

In older adults, limited community ambulation is a risk factor for mobility and self-care decline, decreased health-related quality of life, increased social isolation, and is a marker of frailty [2–5]. Walking in the community is associated with better self-rated health and a lower mortality risk [6–10], but the frequency of community ambulation often decreases with increasing age [11] and can be further negatively impacted when an individual has a chronic health condition, such as osteoarthritis (OA). People with OA in hips or knees may experience joint pain, stiffness, swelling, instability and dysfunction which can restrict a person’s mobility outside of the home [12, 13]. Having hip or knee OA and a self-reported outdoor walking difficulty is associated with a greater mortality risk, compared to those without a reported walking difficulty [9]. Twenty-eight percent of Canadians aged 65–69 years and 47% of Canadians aged 80–84 years live with OA, while 6% of adults aged 45–49 have OA; women have a higher prevalence of OA than men [14]. In the United States, it is estimated that approximately 10% of adults aged 45 and older have OA of the hip and 16% have OA of the knee [15].

Despite the importance of maintaining community ambulation for older adults and people with OA, many individuals do not regularly walk in the community. In Canadian women and men aged 65 to 85, 62.9 and 69.8% walk outside of their home or yard three or more days a week, respectively [5]. This means that approximately 1/3 of older adults do not walk outside regularly [5]. In American adults aged 45 and over with arthritis, 72% described either a lot or a little limitation in walking farther than one mile [16].

In older adults, numerous variables associated with community ambulation have been evaluated across different studies, however, it does not appear that all variables have been combined together. Aspects of walking capacity such as gait speed, endurance and the ability to change postures (i.e., sit to stand, stepping sideways, or backwards) are considered to be important to community ambulation. These aspects are related to safely crossing a street in the time that a walk signal allows and walking distances required to complete necessary activities such as shopping [17–19]. Additional attributes associated with difficulties in walking outdoors for older adults include fear of moving outdoors [2], and low self-efficacy related to community mobility gait, balance, and overcoming barriers [20, 21]. Poor mental health (including depression, stress and emotional problems) is associated with less frequent walking in one’s neighbourhood [22].

For individuals with OA, various factors to date have also been associated with community ambulation, such as: neighbourhood safety [23], knee pain severity, comorbidities, degree of walking limitation, perceived need for walking aids and assistance, and access to a car/public transportation [13]. Canadian adults age 45 and over with OA of the lower extremity are less likely to walk in the community if they have lower endurance, lower self-rated health, severe pain, and are female; and more likely to walk outside if they have fewer chronic health conditions, the weather is warmer, and if they are younger [24].

As noted above, multiple studies have evaluated variables associated with community ambulation for older adults and people with OA. A model that combines multiple variables from the various studies will be informative to identify how these factors are associated with community ambulation and how they are interconnected. Given the importance and health benefits of community ambulation and the fact that many older adults and people with OA of the hip and knee have difficulties walking in the community, modeling community ambulation will provide additional insight into these relationships.

**Fig. 1:**
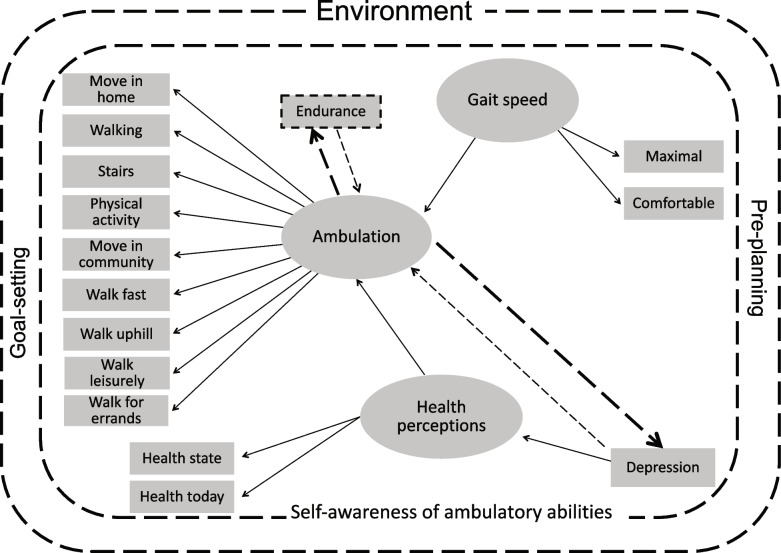
Community ambulation post stroke model. From Barclay R, Ripat J, Mayo N (2015). Factors describing community ambulation after stroke – a mixed-methods study. Clinical Rehabilitation. 29 (5):509–21. Note - Dotted lines were added to the original structural equation model from focus group discussions

A model of community ambulation for people after stroke that used multiple variables of self-report and observed physical function was previously developed [25]. The model is unique in that is was developed using structural equation modeling (SEM) and further refined based on the lived experience of community dwelling stroke survivors who walked in the community [25]. Latent factors in the model include: ambulation (moving in the home, moving in the community, stairs, walking leisurely, walking for exercise, walking uphill, walking for errands), health perceptions, and gait speed. Additional components in the model include depression, endurance, self-awareness of ambulatory ability, goal setting / pre-planning, and the environment [25]. See Fig. 1.

The model focusses on individuals with stroke, however, relationships among these latent factors of community ambulation in older adults and individuals with OA of the hip or knee may be similar and should be examined in these populations, to enable a broader use of the model. Using data from a large population-based dataset with multiple self-report and observed physical function variables will aid in verifying the model of community ambulation in new older adult and OA populations.

In a clinical setting, rehabilitation professionals can use a model of community ambulation to assist in guiding aspects of assessment, intervention, and aid in setting client-centred goals related to walking in the community. Self-reported and observed functioning and health are both important to consider in designing and evaluating a client-specific intervention program that addresses community ambulation. In research, investigators use models to influence the choice of outcome measurement in community ambulation trials and to guide design of new interventions.

The aim of this study was to verify the model of community ambulation post-stroke for older adults and individuals with OA. The first objective was to estimate the associations between the latent constructs of ambulation, gait speed and health perceptions; and variables of depression, sex, age and the environment in older adults aged 65 and older. The second objective was to estimate the associations between the latent constructs of ambulation, gait speed and health perceptions; and variables of depression, sex, age and the environment in adults with osteoarthritis of the hip or knee, aged 45 and older.

## Methods

The CLSA is a longitudinal, population-based study of more than 50,000 Canadians who are being followed for at least 20 years; participants were aged 45–85 at baseline [26, 27]. The CLSA used a stratified random sample. Exclusions to the CLSA at the time of recruitment were: people living in long-term care, people unable to communicate in English or French, people with cognitive impairments, individuals who were Canadian Forces members (full time), people living on Federal First Nation reserves and First Nation settlements, or those living in any of Canada’s three Territories [26, 27]. The CLSA consists of two cohorts: the Tracking cohort and the Comprehensive cohort.

### Study sample

This study used data from the CLSA Baseline Comprehensive Dataset version 4.0, (*n* = 30,097), which utilized the following sources: 1) In-Home Questionnaire - baseline; 2) Data Collection Site Questionnaire; 3) Physical Assessments at Data Collection Site; and 4) Maintaining Contact Questionnaire (Wave 1 Version). Data were collected beween 2010 and 2015 [28]. This study, a secondary analysis of the CLSA dataset, received formal approval from the Health Research Ethics Board, University of Manitoba; HS22810 (H2019:173).

To identify which participants were older adults, aged 65 and older, the age at the baseline interview was utilized. To identify the participants with OA in the lower extremity, specific items in the baseline interview were used. Interviewers asked each participant during the baseline interview, “Has a doctor ever told you that you have osteoarthritis in the knee?” and “Has a doctor ever told you that you have osteoarthritis in the hip?” [29]. If the answer to either question was ‘yes’, the participant was identified as having OA in the lower extremity for the purpose of this study.

#### Missing data and sample size

Participants who reported not being able to walk (*n* = 51) were removed from the dataset, as the focus was on ambulation in the community. In the current study, we focus on two models, using data from those aged 65–85 (referred to as 65+) and those with OA aged 45–85. The 65+ group consisted of 12,625 participants while the OA group consisted of 5930 participants.

Ordinal variables in the measurement models were recoded to let higher values indicate better circumstances. An exception was for the covariate of depression where higher scores were equal to higher depression. Observed physical tests (continuous variables) retained their scoring direction (described in Table 1).

**Table 1:** Community Ambulation model – items proposed for initial models

Latent factor	Variable	Label on models	Original source^a^	Scoring meaning	Description or question wording
**Ambulation**	Moving outside of bedroom- help^b^	Room aid	LSI	Higher score = less use of assistance 4 categories	“Did you use aids or equipment, or need help from another person to get to other rooms of your home besides the room where you sleep?” (Combined with Y/N- have you been to other rooms…)
	Moving outside of bedroom- frequency^b^	Room freq	LSI	Higher score = more frequent 5 categories	“How often did you get to other rooms of your home besides the room where you sleep?” (Combined with Y/N)
	Moving outside of home - help	Out aid	LSI	Higher score = less use of assistance 4 categories	“Did you use aids or equipment, or need help from another person to get to an area outside your home such as your porch, deck or patio, hallway (of an apartment building) or garage, in your own yard or driveway?” (Combined with Y/N)
	Moving outside of home - frequency	Out freq	LSI	Higher score = more frequent 5 categories	“How often did you get to an area outside your home such as your porch, deck or patio, hallway (of an apartment building) or garage, in your own yard or driveway?” (Combined with Y/N)
	Moving in neighbourhood - help	Neighbourhood aid	LSI	Higher score = less use of assistance 4 categories	“Did you use aids or equipment, or need help from another person to get to places in your neighbourhood, other than your own yard or apartment building?” (Combined with Y/N)
	Moving in neighbourhood - frequency	Neighbourhood freq	LSI	Higher score = more frequent 5 categories	“How often did you get to places in your neighbourhood, other than your own yard or apartment building?” (Combined with Y/N)
	Moving in town - help	Town aid	LSI	Higher score = less use of assistance 4 categories	“Did you use aids or equipment, or need help from another person to get to places outside your neighbourhood, but within your town?” (Combined with Y/N)
	Moving in town - frequency	Town freq	LSI	Higher score = more frequent 5 categories	“How often did you get to places outside your neighbourhood, but within your town?” (Combined with Y/N)
	Walking ability	Walk help	OARS (walking ability)	Higher score = no help 2 categories	Able to walk with help, or able to walk without help. Help = help of a person or mobility aid (Combined variables of walking aids required plus walking ability)
	Walking outdoors frequency	Walk outdoors	PASE	Higher score = more frequently walking outdoors 4 categories	“Over the past 7 days, how often did you take a walk outside your home or yard for any reason? For example, for pleasure or exercise, walking to work, walking the dog, etc.”
	Walking endurance	Endurance	PASE	Higher score = more walking 6 categories	Hours per day spent walking
**Health perception**	General health	Gen health		Higher score = higher self-rated health 5 categories	“In general, would you say your health is excellent, very good, good, fair, or poor?”
	Pain preventing activities	Pain prevent		Higher score = less activities prevented by pain 4 categories	“How many activities does your pain or discomfort prevent? Would you say none, a few, some, or most?”
	Pain intensity	Pain intensity		Higher score = less pain 4 categories	“How would you describe the usual intensity of your pain or discomfort? Would you say it is mild, moderate, or severe?” combined with “Are you usually free of pain or discomfort?”
**Timed Functional mobility**	Gait speed	Gait speed	Four-metre Walk Test	Higher score = faster continuous	Time to walk 4 m expressed as gait speed (metres/second) Contraindication - unable to stand or walk without the assistance of another person
	Functional speed	TUG	Timed Up and Go	Higher score = slower, less independent continuous	Total time in seconds required to stand up, walk 3 m, turn around and sit back down - Timed Up and Go test (in seconds) Contraindications - unable to stand without the assistance of another person, unable to rise from a chair without the assistance of another person, unable to walk without the assistance of another person
	Standing up - Leg strength	STS	Chair Rise	Higher score = slower, more difficult continuous	Total time required to completely stand up and sit down from chair 5 times (in seconds) Contraindication - unable to stand or rise from a chair unassisted
	Balance	Balance	Standing balance test	Higher score = better balance continuous	Best attained time for standing on one leg (in seconds) Contraindication - unable to stand unassisted
**Environment**	Fear in walking alone after dark^c^	Fear	HRS	Higher score = lower fear 4 categories	Response to “People would be afraid to walk alone after dark in this area”
	Living in urban or rural setting^d^	Urban		2 categories	Five urban classifications combined as ‘urban’ and one rural category
**Covariates in structural model:**	Depression frequency	Depression	CES-D 10	Higher score, higher depression 4 categories	“How often did you feel depressed?” (in the last week)
	Sex	Sex		0 – Female 1 - Male	Sex
	Age	Age		74–86 65–74 55–64 45–54	Age groups by 10 year age groups
	Frequency of falls	Falls		0–20 continuous	Number of Falls in past 12 months

^a^ see text for references

^b^ variables not in final OA model

^c^ variable treated as a covariate in final OA and 65–86 year models

^d^ variable not in final OA and 65–86 year models

*LSI* Life Space Index, *OARS* Older Americans Resources and Services, *PASE* Physical Activity Scale for the Elderly, *CES-D 10* Center for Epidemiologic Studies Short Depression Scale, *HRS* Health and Retirement Survey, *Y/N* yes / no

For categorical variables, “Don’t know”, “No Answer”, and “Refused” were treated as missing. For all the categorical variables in the model, the percent of Don’t know/refused was less than 3%, with the majority of the variables less than 1%. Observations that had extreme values in chair rise time (> = 129 seconds, 1 observation in 65+ group), TUG time (> 60 seconds, 5 observations in 65+ group), number of falls in the previous 12 months (> = 24, 4 observations in 65+ group, 1 observation in OA group) were recoded as missing. The percent of missing in all the variables in this study after the missingness recoding described above was less than 10%, except for timed balance in the OA group (10.3% missing). Missingness is higher in the observed physical tests than the self-report tests, likely due to the multiple contra-indications for the physical tests, as outlined in Table 1.

While there is no agreed upon recommendation for sample size for SEM, it has been recommended that 20 participants are required for each estimated parameter in SEM [30]. The sample size of the CLSA dataset and the groupings used were therefore deemed sufficient for SEM analyses.

### Items and outcomes measures

We used multiple items to develop the models for age 65+ and those with OA. Many items in the CLSA come from common outcome measures with evidence of reliability and validity [27]. For example, items included were from measures such as the Life Space Index (LSI) [31], Older Americans Resources and Services (OARS) [32], Physical Activity Scale for the Elderly (PASE) [33], Center for Epidemiologic Studies Short Depression Scale (CES-D 10) [34], Health and Retirement Survey [27] and commonly used self-rated health and pain items from previous studies [27].

Physical function items included the timed up and go (TUG) [35], standing balance test [36], four-metre walk test (to determine gait speed) [37], and sit to stand (STS) chair rise as a representation of leg strength [38]. Please see Table 1 for a summary of items proposed for the initial models with brief descriptions of each item. Additional information is also available from the CLSA protocol [27].

### Statistical analysis

All the statistical analyses were conducted in R 3.6.3 for Windows. The “lavaan” package in R was used to perform SEM analyses. All analyses used unweighted data; we did not use the CLSA sample weights, since the “lavaan” package does not allow sample weights with the weighted least square mean and variance (WLSMV) estimation method used in the SEM analysis.

SEM is a statistical approach used to examine the complex relationship of latent constructs and observed variables [39]. It consists of measurement models that represent the way of measuring latent constructs by a number of observed variables and a structural model that represents relationships among the latent constructs and observed variables. The observed variables in a measurement model are commonly called indicators or items, while the observed variables in a structural model are commonly called covariates [30]. The latent constructs are called latent factors. Factor loadings indicate the path coefficients from latent construct to indicators; the square of a standardized factor loading reflects the percent of variation in the indicator that can be captured by the latent factor [30].

The WLSMV estimator was used to estimate parameters in SEMs with ordinal variables [40, 41]. Delta parameterization was used to fix the total variance of the latent factors to one in order to make the models identified [30]. Missing data of indicators were addressed by pairwise deletion [42]. Missing data of covariates in the structural model were addressed by listwise deletion. The full information maximum likelihood (ML) method was not used in this study because this method was not supported when using WLSMV approach in the “lavaan” package. The performance of measurement models and SEMs were assessed by the robust chi-square (*χ*^2^) test, robust comparative fit index (CFI), and robust root mean square error of approximation (RMSEA) with its 90% confidence interval (CI) [30, 43]. For simplicity, the word robust was omitted in the following text. Non-significant *χ*^2^ test results, CFI larger than 0.9, or RMSEA less than 0.08 indicates that the model has sufficient fit to the data [30].

Specifically, RMSEA of ≤0.05 suggests a close fit and RMSEA of 0.05–0.08, a reasonable fit [30]. The *χ*^2^ test is sensitive to sample size, therefore, more weight should be given to other goodness-of-fit indices [30]. Although the robust version of CFI and RMSEA are developed when using the WLSMV estimator, they could be problematic when using the conventional cutoffs developed for ML for continuous data [43, 44]. Therefore, the standardized root mean square residual (SRMR), which has been recommended when using the WLSMV estimator and the conventional cutoff (i.e., ≤ 0.09) [45–48], was also used to assess model fit of SEMs.

Descriptive statistics were generated for the two study groups (65+ and OA), including mean, standard deviation (SD) and range for continuous variables as well as frequencies and percentages for categorical variables. Gait speed (metres/second) and the best time in seconds attained in standing balance were rescaled through multiplying by 10 and dividing by 10, respectively. To check correlations among observed variables and to identify collinearity, correlation-coefficient matrices were calculated: polychoric correlation coefficient for pairs of ordinal variables, polyserial for pairs of ordinal and continuous variables, and Pearson for pairs of continuous variables [30]. See Supplementary Table S1 (65+ group) and Table S2 (OA group).

**Fig. 2:**
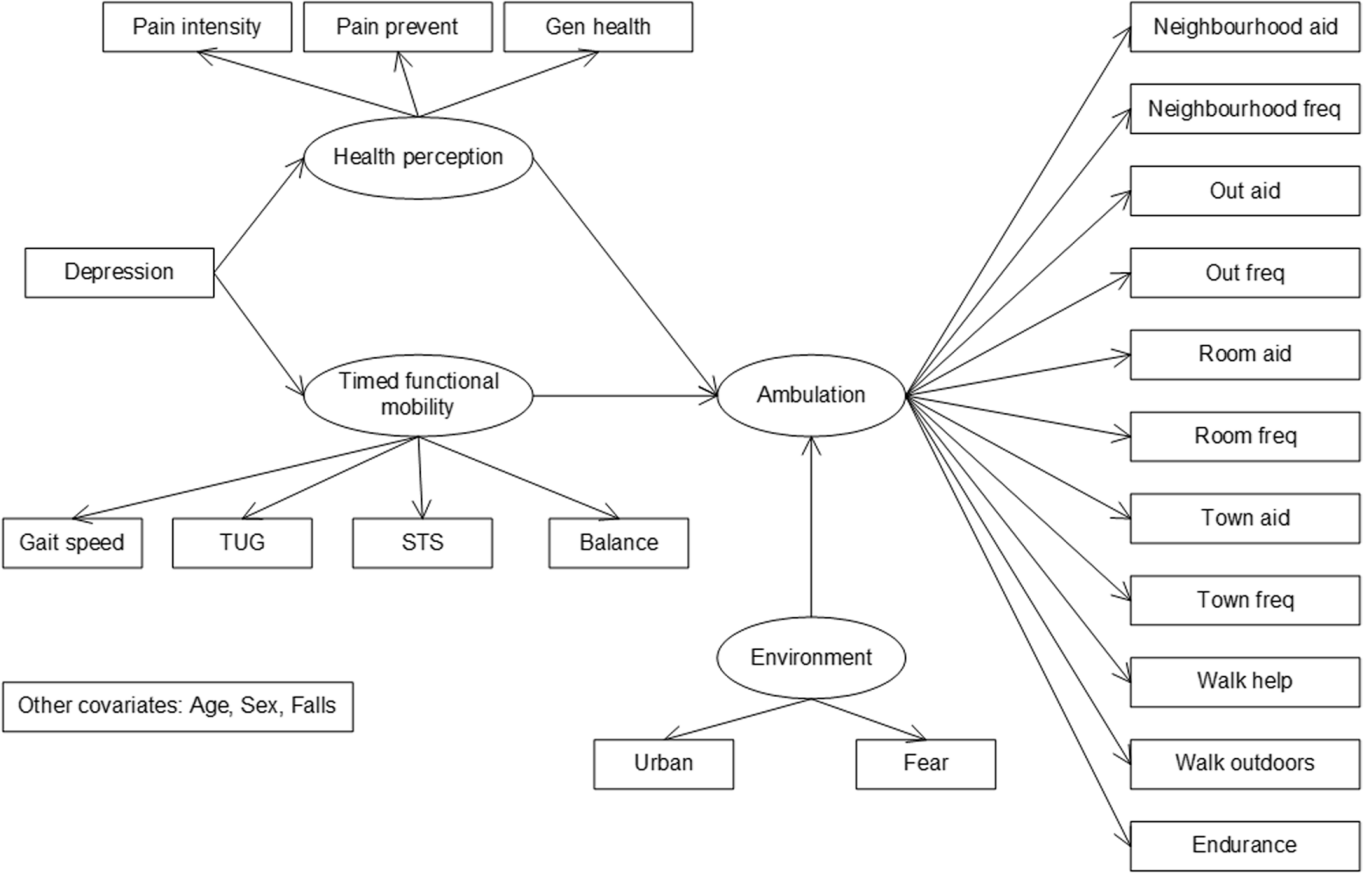
Initial SEM implied by the model developed for people after stroke

The initial SEM for this project, implied by the model developed to describe community ambulation after stroke [25] is shown in Fig. 2. The four latent factors in the initial SEM were ambulation (11 indicators), health perceptions (3 indicators), timed functional mobility (4 indicators), and environment (2 indicators). The CLSA has multiple timed physical variables related to walking; therefore, we chose to substitute the original latent factor ‘gait speed’ with ‘timed functional mobility’. In a study of 75 year old women (*n* = 230), timed balance standing on one leg was correlated with timed gait performance and with isometric knee extension; timed gait performance was associated with isometric knee flexion and extension and ankle dorsiflexion [49]. It was reasonable therefore, to include timed STS (leg strength), TUG, gait speed and timed balance on one leg in a ‘timed functional mobility’ latent factor. The direction of the path from depression to health perception was maintained from the original model.

The original post-stroke model included endurance, aspects of the environment and the concepts of goal setting, self-awareness and pre-planning. The concepts were added to the SEM after focus group discussions with stroke survivors [25]. We were able to include endurance in the ambulation latent factor as well as an environment latent factor. We were not, however able to include goal setting, self-awareness or pre-planning, based on the variables available in the dataset.

Measurement models for latent constructs were investigated first to check if latent factors were properly constructed. Modifications of measurement models were made based on results and knowledge of the literature. Then, the modified measurement models and covariates were included in the structural model. Similarly, modifications to the SEM were based on the modification index [42] and knowledge in the literature. Equivalent or near equivalent models of the final SEM were tested and compared to determine our final model. A final model for the 65+ group was developed first and the OA model was developed after the 65+ model.

Measurement invariance for sex was tested for the 65+ and OA models using multi-group SEM to investigate if the latent factors were measured in the same way for males and females. Measurement invariance for age (65+ and < 65) was also tested in the OA model. Three levels of measurement invariance (configural, metric, and scalar invariance) were tested through comparing hierarchically more constrained models with less constrained ones. The latent constructs have the same structure of measurement models across groups if configural invariance is retained; metric invariance is supported if the model with factor loadings constrained to be equivalent does not have significantly worse fit than the model without this constraint; scalar invariance is retained if the model with factor loadings and intercept of indicators constrained to be equivalent does not have significant worse fit than the metric invariance model [50]. The likelihood ratio test based on robust *χ*^2^ statistics, *χ*^2^ difference ( ∆ χ^2^) and CFI difference (Δ CFI) were used to compare the hierarchical models [51]. A non-significant result of ∆*χ*^2^ or Δ CFI < 0.01 supports that the invariance is retained. However, more weight should be given to Δ CFI as the *χ*^2^ statistics are sensitive to sample size [51]. Valid comparison of group means on the latent factors can be achieved once the configural, metric and scalar invariance are supported [50, 52].

## Results

In the 65+ group and OA group, there were 12,625 and 5930 participants. Participants who had complete data for the SEM covariates of age group, sex, depression, and fear in walking alone were included in the statistical analyses: 11,619 in the 65+ group and 5546 in the OA group. Fifty-six percent (*n* = 3111) of the OA group were 65 years of age or older and 26.5% of the 65+ group had OA.

### 65+ group participant characteristics

The mean age in the 65+ group was 73 ± 6, ranging from 65 to 85; 41% were older than 75 years. See Table 2 for the characteristics of the participants aged 65+. Almost half of the participants in the 65+ group were female (49%), 64% were married or living with a common-law partner, 93% were living in urban areas, 72% had a post-secondary degree or diploma, and 55% had a household income of $50,000 or more. A majority of the 65+ group (92%) had two or more chronic conditions. General health was rated as very good or excellent by 62% of the 65+ group, and 65% spent an average of 30 minutes or more walking per day. A majority of those 65+ did not need an aid to move to other rooms or outside their home, while 5% needed help (personal or equipment) to move in the neighbourhood.

**Table 2:** Characteristics of older adult participants, aged 65+

Characteristic	Categories/units	n	%	missing n / %	mean ± SD (range)
Age	Years	11,619	100%	0/0.0	73 ± 6 (65,86)
Age group	65–74	6910	59%	0/0.0	
	75+	4709	41%		
Sex	Male	5903	51%	0/0.0	
	Female	5716	49%		
Marital status	Single, never married or never lived with a partner	656	6%	1/0.0	
	Married/Living with a partner in a common-law relationship	7378	64%		
	Widowed	2042	18%		
	Divorced	1333	12%		
	Separated	209	2%		
Education	Less than secondary school graduation	1004	9%	32/0.3	
	Secondary school graduation, no post-secondary education	1243	11%		
	Some post-secondary education	954	8%		
	Post-secondary degree/diploma	8386	72%		
Total household income	Less than $20,000	675	6%	1014/8.7	
	$20,000 or more, but less than $50,000	3524	30%		
	$50,000 or more, but less than $100,000	4254	37%		
	$100,000 or more, but less than $150,000	1428	12%		
	$150,000 or more	724	6%		
Number chronic conditions	0	238	2%	0/0.0	
	1	692	6%		
	2	1202	10%		
	3	1557	13%		
	4	1601	14%		
	5	1599	14%		
	6	1341	12%		
	7+	3389	29%		
Urban/Rural	Urban	10,824	93%	0/0.0	
	Rural	795	7%		
Depression	Rarely or never (less than 1 day)	9597	83%	0/0.0	
	Some of the time (1–2 days)	1284	11%		
	Occasionally (3–4 days)	559	5%		
	All of the time (5-7 days)	179	2%		
Fear in walking alone after dark	Strongly agree	171	2%	0/0.0	
	Agree	1331	12%		
	Disagree	6866	59%		
	Strongly disagree	3251	28%		
Frequency of falls	# of falls in the past 12 months	11,600	100%	19/0.2	0.2 ± 0.7 (0, 20)
Moving in neighbourhood - help	NOT BEEN to places in your neighbourhood other than your own yard or driveway	200	2%	2/0.0	
	Yes, personal assistance	58	1%		
	Yes, equipment only	500	4%		
	No	10,859	93%		
Moving in neighbourhood - frequency	Never	200	2%	4/0.0	
	Less than once per week	117	1%		
	1 to 3 times per week	1240	11%		
	4 to 6 times per week	2526	22%		
	Daily	7532	65%		
Moving outside of home - help	NOT BEEN to places outside your home such as your porch, deck or patio, hallway (of an apartment building) or garage, in your own yard or driveway	23	0%	1/0.0	
	Yes, personal assistance	17	0%		
	Yes, equipment only	352	3%		
	No	11,226	97%		
Moving outside of home - frequency	Never	23	0%	6/0.0	
	Less than once per week	26	0%		
	1 to 3 times per week	237	2%		
	4 to 6 times per week	807	7%		
	Daily	10,520	91%		
Moving outside of bedroom- help	NOT BEEN to other rooms of your home besides the room where you sleep	22	0%	1/0.0	
	Yes, personal assistance	6	0%		
	Yes, equipment only	226	2%		
	No	11,364	98%		
Moving outside of bedroom- frequency	Never	22	0%	3/0.0	
	Less than once per week	5	0%		
	1 to 3 times per week	3	0%		
	4 to 6 times per week	16	0%		
	Daily	11,570	100%		
Moving in town - help	NOT BEEN to places outside your neighbourhood, but within your town	72	1%	2/0.0	
	Yes, personal assistance	126	1%		
	Yes, equipment only	511	4%		
	No	10,908	94%		
Moving in town - frequency	Never	72	1%	6/0.0	
	Less than once per week	301	3%		
	1 to 3 times per week	3386	29%		
	4 to 6 times per week	3933	34%		
	Daily	3921	34%		
Walking ability	Walk with help of person or used one or more of the mobility aids (cane, wheelchair, scooter, walker, leg braces)	1827	16%	10/0.1	
	Walk without help	9782	84%		
Walking outdoors frequency	Never	1992	17%	17/0.1	
	Seldom (1 to 2 days)	1605	14%		
	Sometimes (3 to 4 days)	2082	18%		
	Often (5 to 7 days)	5923	51%		
Walking endurance	Never	1992	17%	69/0.5	
	Less than 30 minutes	1992	17%		
	30 minutes but less than 1 hour	4182	36%		
	1 hour but less than 2 hours	2614	22%		
	2 hours but less than 4 hours	648	6%		
	4 hours or more	122	1%		
Pain intensity	Severe usual	479	4%	97/0.8	
	Moderate	2243	19%		
	Mild	1756	15%		
	Usually free	7044	61%		
Pain preventing activities	NOT free of pain, MOST activities prevented by pain or discomfort	444	4%	68/0.5	
	NOT free of pain, SOME activities prevented by pain or discomfort	820	7%		
	NOT free of pain, a FEW activities prevented by pain or discomfort	1351	12%		
	Free of pain or no activities prevented by pain or discomfort	8936	77%		
General health	Poor	150	1%	12/0.1	
	Fair	861	7%		
	Good	3483	30%		
	Very good	4777	42%		
	Excellent	2336	20%		
Balance	Best attained time for standing on one leg (in seconds)	10,695	92%	924/8.0	22.1 ± 21.9 (0.0,60.0)
Standing up - Leg strength	Seconds to completely stand up and sit down from chair 5 times	10,936	94%	683/5.9	14.2 ± 3.9 (2.1,60.0)
Gait speed	metres/second	11,469	99%	150/1.3	0.9 ± 0.2 (0.2,2.3)
Functional speed (TUG)	Seconds required to stand up, walk 3 m, turn around, return and sit	11,446	99%	173/1.5	10.3 ± 2.6 (2.8,48.5)

#### Goodness-of-fit of SEM for 65+ group

The measurement models for ambulation ((*χ*^2^ = 1299.65, degrees of freedom (df) = 41, CFI = 0.98, RMSEA (90% CI) = 0.051 (0.049–0.054)), and timed functional mobility ((*χ*^2^ = 97.36, df = 2, CFI = 0.99, RMSEA (90% CI) = 0.064 (0.056–0.079)) had reasonable fit to the data. These two measurement models, along with the health perceptions latent factor with three indicators and the environment latent factor with two indicators were included in the SEM. The initial SEM had a close goodness-of-fit ((*χ*^2^ = 2297.13, df = 158, CFI = 0.98, RMSEA (90% CI) = 0.034 (0.033–0.035), SRMR = 0.08). However, the standardized factor loading from the environment latent factor to urban/rural was close to zero although the standardized factor loading from the environment latent factor to fear in walking alone after dark was 0.66. Moreover, the correlation coefficient between urban/rural and fear in walking alone after dark was close to zero (see Supplementary Table S1). Therefore, instead of assuming an environment latent factor, we treated the variable, fear in walking alone after dark, as one covariate in the structural model. A high score on this variable equals low fear. This model had better goodness-of-fit indices ((*χ*^2^ = 1051.78, df = 142, CFI = 0.98, RMSEA (90% CI) = 0.023 (0.022–0.025), SRMR = 0.09). When depression was added as a covariate to the model, there was a close fit ((*χ*^2^ = 1169.57, df = 158, CFI = 0.97, RMSEA (90% CI) = 0.023 (0.022–0.025), SRMR = 0.09) to the data.

Then, other covariates (age group, sex, and number of falls) were added to the structural model according to the literature and modification indices, one by one. The final SEM had a close goodness-of-fit ((*χ*^2^ = 983.46, df = 208, CFI = 0.91, RMSEA (90% CI) = 0.018 (0.017–0.019), SRMR = 0.09).

The final model had significant better fit to data than the two equivalent or near equivalent models (Supplementary Table S3), which had the opposite arrow direction of falls and timed functional mobility or removed this path. Another alternate model involves the covariate of fear in walking alone after dark. In the final model, the path from fear of walking after dark to timed functional mobility (which includes gait speed) was added based on a modification index and team discussion that this could be feasible. It has been suggested that fear of falling influences gait speed in older adults [53], so it appeared possible that fear of walking outdoors after dark could have a similar effect. It has also been identified that there is an association between fear of moving outdoors and slower gait speed [2]. This path could potentially also be in the opposite direction, from timed functional mobility to fear. We evaluated that alternative model and it also had acceptable fit (*χ*^2^ =1356.01 (211), CFI = 0.93, RMSEA = 0.022 (0.021, 0.023), SRMR = 0.09) and the path from fear to ambulation became insignificant. See Supplementary Fig. S1 for the alternative model in the 65+ group. The final model was chosen as it had a lower RMSEA compared to the alternate model.

**Fig. 3:**
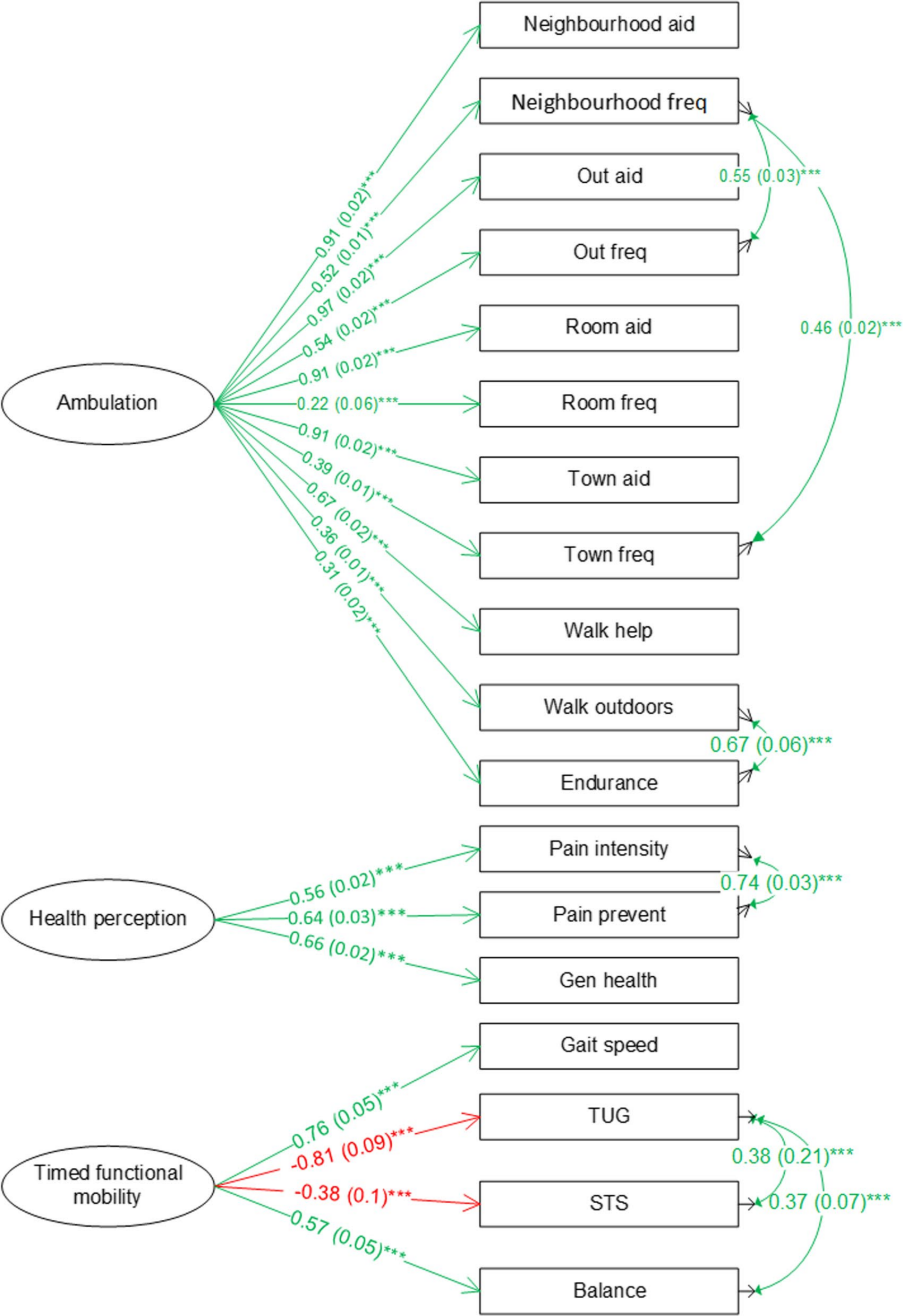
Measurement models of the final community ambulation SEM for 65+ group. Note: green indicates positive association; red indicates negative association; cell format: standardized factor loading (standard error) ^significance level^ *** p < 0.001

The measurement models which are part of the final SEM are shown in Fig. 3. All the standardized factor loadings from ambulation to its indicators were positive indicating that the latent construct ambulation represented higher levels of ambulation. Similarly, health perceptions also represented better conditions. The positive standardized factor loadings from the timed functional mobility latent factor to gait speed and balance, and negative standardized factor loadings to TUG and STS (leg strength) times supported that timed functional mobility represented better functional mobility.

**Fig. 4:**
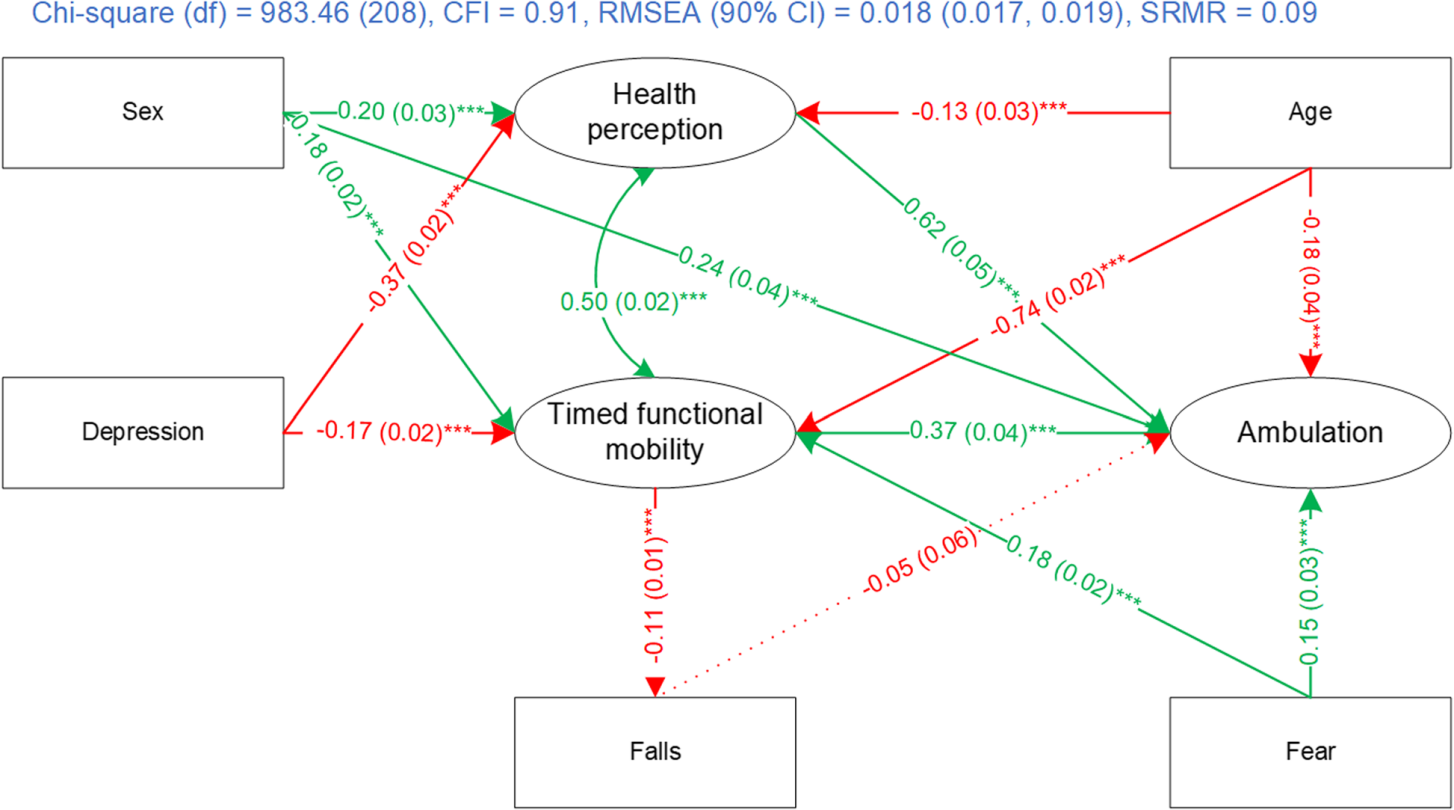
Structural model of the final community ambulation model for 65+ group. Note: green indicates positive association; red indicates negative association; cell format: path coefficient (standard error) ^significance level^ Path coefficients are not standardized. *** p < 0.001. Chi-square = robust chi-square test statistics, df = degree of freedom, CFI = robust comparative fit index, RMSEA = robust root mean square error of approximation, SRMR = standardized root mean residual

The structural model in the final SEM is shown in Fig. 4. Health perceptions (path coefficient = 0.62, *p* < .001) and timed functional mobility (path coefficient = 0.37, *p* < .001) were positively and significantly associated with ambulation. Depression was negatively associated with health perceptions (path coefficient = − 0.37, *p* < .001) and timed functional mobility (path coefficient = − 0.17, *p* < .001). Males had better health perceptions, timed functional mobility, and ambulation according to the positive path coefficients (path coefficients = 0.2, *p* < .001; 0.18, *p* < .001; 0.24, *p* < .001). Age group was negatively associated with health perceptions (path coefficient = − 0.13, *p* < .001), timed functional mobility (path coefficient = − 0.74, *p* < .001) and ambulation (path coefficient = − 0.18, *p* < .001) indicating that older adults in the older age group (75+) had lower health perceptions and worse timed functional mobility and ambulation. Timed functional mobility was negatively associated with number of falls (path coefficient = − 0.11, *p* < .001) while number of falls (path coefficient = − 0.05, NS) was negatively associated with ambulation, though not statistically significant. Feeling comfortable walking after dark in one’s neighbourhood was positively related to timed functional mobility and ambulation.

Measurement invariance from configural to scalar invariance was maintained across male and female groups. Therefore, the comparison of means of latent constructs (i.e., health perceptions, timed functional mobility, and ambulation) across males and females is valid. See Table 3.

**Table 3:** Measurement invariance for sex (males vs. females) for the 65+ group

Invariance Type	*χ*^2^ (df)	∆ *χ*^2^ (∆df)	RMSEA	CFI	∆ CFI
Configural	1252.49 (384)		0.02	0.977	
Metric	1217.24 (399)	−35.25 (15)	0.019	0.978	< 0.01
Scalar	1222.73 (431)	5.49 (32)	0.018	0.979	< 0.01

***χ***^**2**^ *= robust* ***χ***^**2**^ *test statistics, df* degree of freedom, *CFI robust* comparative fit index, *RMSEA robust* root mean square error of approximation, **∆ =** *change.*

### OA group participant characteristics

The mean age in the OA group was 67 ± 10, ranging from 45 to 86; 44% were younger than 65. See Table 4 for characteristics of the participants with OA. Sixty percent of those with OA were female, 64% were married or living with common-law partner, 93% were living in urban areas, 75% had a post-secondary degree or diploma, and 61% had household income of $50,000 CDN or more. Almost all participants with OA (98%) had two or more chronic conditions. More than half (52%) of the OA group rated their general health as very good or excellent, and 62% spent 30 minutes or more walking per day. A majority of those with OA did not need an aid to move to other rooms or outside their home, while about 8% needed assistance (personal or equipment) to move in their neighbourhood.

**Table 4:** Characteristics of participants with osteoarthritis of hip and/or knee

Characteristic	Categories/units	n	%	missing n/%	mean ± SD (range)
Age	Years	5546	100%	0/0.0	67 ± 10 (45, 86)
Age group	45–54	660	12%	0/0.0	
	55–64	1775	32%		
	65–74	1754	32%		
	75+	1357	24%		
Sex	Male	2233	40%	0/0.0	
	Female	3313	60%		
Marital status	Single, never married or never lived with a partner	482	9%	3/0.1	
	Married/Living with a partner in a common-law relationship	3538	64%		
	Widowed	737	13%		
	Divorced	663	12%		
	Separated	123	2%		
Education	Less than secondary school graduation	394	7%	10/0.2	
	Secondary school graduation, no post-secondary education	525	9%		
	Some post-secondary education	442	8%		
	Post-secondary degree/diploma	4175	75%		
Total household income	Less than $20,000	352	6%	422/7.6	
	$20,000 or more, but less than $50,000	1396	25%		
	$50,000 or more, but less than $100,000	1918	35%		
	$100,000 or more, but less than $150,000	833	15%		
	$150,000 or more	625	11%		
Number chronic conditions	1	113	2%	0/0.0	
	2	304	5%		
	3	507	9%		
	4	635	11%		
	5	746	13%		
	6	754	14%		
	7+	2487	45%		
Urban/Rural	Urban	5152	93%	0/0.0	
	Rural	394	7%		
Depression	Rarely or never (less than 1 day)	4240	77%	0/0.0	
	Some of the time (1–2 days)	779	14%		
	Occasionally (3–4 days)	388	7%		
	All of the time (5-7 days)	139	3%		
Fear in walking alone after dark	Strongly agree	96	2%	0/0.0	
	Agree	670	12%		
	Disagree	3100	56%		
	Strongly disagree	1680	30%		
Frequency of falls	# of falls in the past 12 months	5538	100%	8/0.1	0.2 ± 0.8 (0, 20)
Moving in neighbourhood - help	NOT BEEN to places in your neighbourhood other than your own yard or driveway	113	2%	1/0.0	
	Yes, personal assistance	35	1%		
	Yes, equipment only	374	7%		
	No	5023	91%		
Moving in neighbourhood - frequency	Never	113	2%	0/0.0	
	Less than once per week	66	1%		
	1 to 3 times per week	592	11%		
	4 to 6 times per week	1170	21%		
	Daily	3605	65%		
Moving outside of home - help	NOT BEEN to places outside your home such as your porch, deck or patio, hallway (of an apartment building) or garage, in your own yard or driveway	14	0%	0/0.0	
	Yes, personal assistance	14	0%		
	Yes, equipment only	261	5%		
	No	5257	95%		
Moving outside of home - frequency	Never	14	0%	1/0.0	
	Less than once per week	15	0%		
	1 to 3 times per week	120	2%		
	4 to 6 times per week	403	7%		
	Daily	4993	90%		
Moving outside of bedroom- help	NOT BEEN to other rooms of your home besides the room where you sleep	15	0%	0/0.0	
	Yes, personal assistance	6	0%		
	Yes, equipment only	167	3%		
	No	5358	96%		
Moving outside of bedroom- frequency	Never	15	0%	3/0.1	
	Less than once per week	0	0%		
	1 to 3 times per week	2	0%		
	4 to 6 times per week	11	0%		
	Daily	5515	99%		
Moving in town - help	NOT BEEN to places outside your neighbourhood, but within your town	39	1%	3/0.1	
	Yes, personal assistance	80	1%		
	Yes, equipment only	388	7%		
	No	5036	91%		
Moving in town - frequency	Never	39	1%	2/0.0	
	Less than once per week	157	3%		
	1 to 3 times per week	1442	26%		
	4 to 6 times per week	1729	31%		
	Daily	2177	39%		
Walking ability	Walk with help of person or used one or more of the mobility aids (cane, wheelchair, scooter, walker, leg braces)	1441	26%	4/0.1	
	Walk without help	4101	74%		
Walking outdoors frequency	Never	1030	19%	6/0.1	
	Seldom (1 to 2 days)	839	15%		
	Sometimes (3 to 4 days)	970	18%		
	Often (5 to 7 days)	2701	49%		
Walking endurance	Never	1030	19%		
	Less than 30 minutes	1043	19%	28/0.5	
	30 minutes but less than 1 hour	1940	35%		
	1 hour but less than 2 hours	1167	21%		
	2 hours but less than 4 hours	276	5%		
	4 hours or more	62	1%		
Pain intensity	Severe usual	377	7%	57/1.0	
	Moderate	1676	30%		
	Mild	1132	20%		
	Usually free	2304	42%		
Pain preventing activities	NOT free of pain, MOST activities prevented by pain or discomfort	402	7%	37/0.7	
	NOT free of pain, SOME activities prevented by pain or discomfort	728	13%		
	NOT free of pain, a FEW activities prevented by pain or discomfort	1052	19%		
	Free of pain or no activities prevented by pain or discomfort	3327	60%		
General health	Poor	135	2%	5/0.1	
	Fair	628	11%		
	Good	1898	34%		
	Very good	2104	38%		
	Excellent	776	14%		
Balance	Best attained time for standing on one leg (in seconds)	4974	90%	572/10.3	27.8 ± 24.1 (0.1,60.0)
Standing up - Leg strength	Seconds to completely stand up and sit down from chair 5 times	5068	91%	478/8.6	14.1 ± 4.1 (2.1, 60.0)
Gait speed	metres/second	5463	99%	83/1.5	0.9 ± 0.2 (0.2, 2.6)
Functional speed (TUG)	Seconds required to stand up, walk 3 m, turn around, return and sit	5448	98%	98/1.8	10.3 ± 2.7 (2.8, 35.8)

#### Goodness-of-fit of SEM for OA group

A similar model building approach was implemented for the OA group. The variables for moving outside of the bedroom were removed from the model because of zero values in one response category. The measurement models for ambulation ((*χ*^2^ = 566.00, df = 24, CFI = 0.99, RMSEA (90% CI) = 0.064 (0.059–0.068)), and timed functional mobility ((*χ*^2^ = 59.00, df = 2, CFI = 0.99, RMSEA (90% CI) = 0.076 (0.06–0.094)) had acceptable fit to the data. Then, these two measurement models, along with the health perceptions latent factor with three indicators and the covariates fear in walking alone after dark and depression, were included in the SEM. This model had close goodness-of-fit ((*χ*^2^ = 724.27, df = 124, CFI = 0.97, RMSEA (90% CI) = 0.030 (0.027–0.032), SRMR = 0.05).

**Fig. 5:**
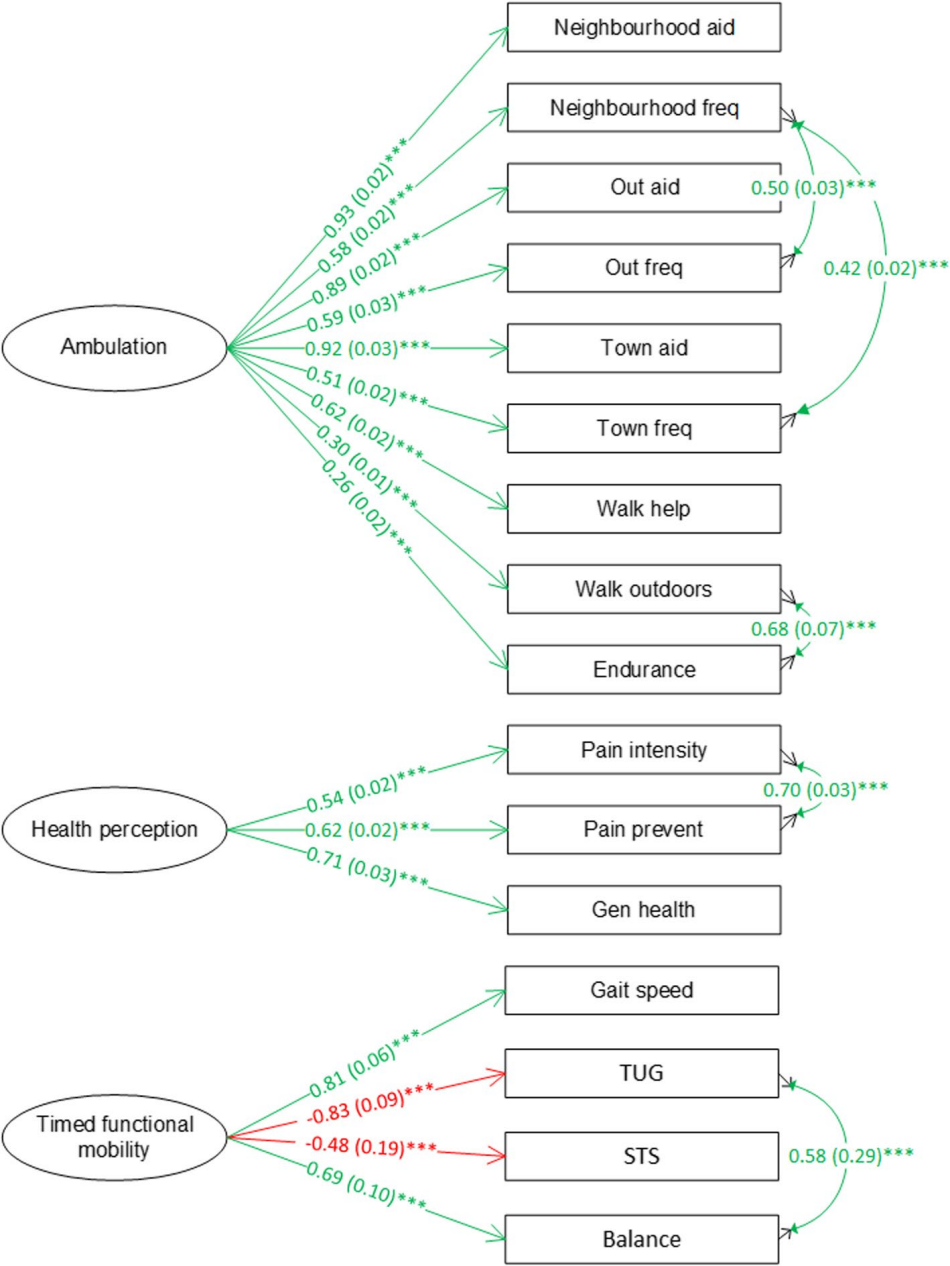
Measurement models of the final community ambulation SEM for OA group. Note: green indicates positive association; red indicates negative association; cell format: standardized factor loading (standard error) ^significance level^, *** p < 0.001

**Fig. 6:**
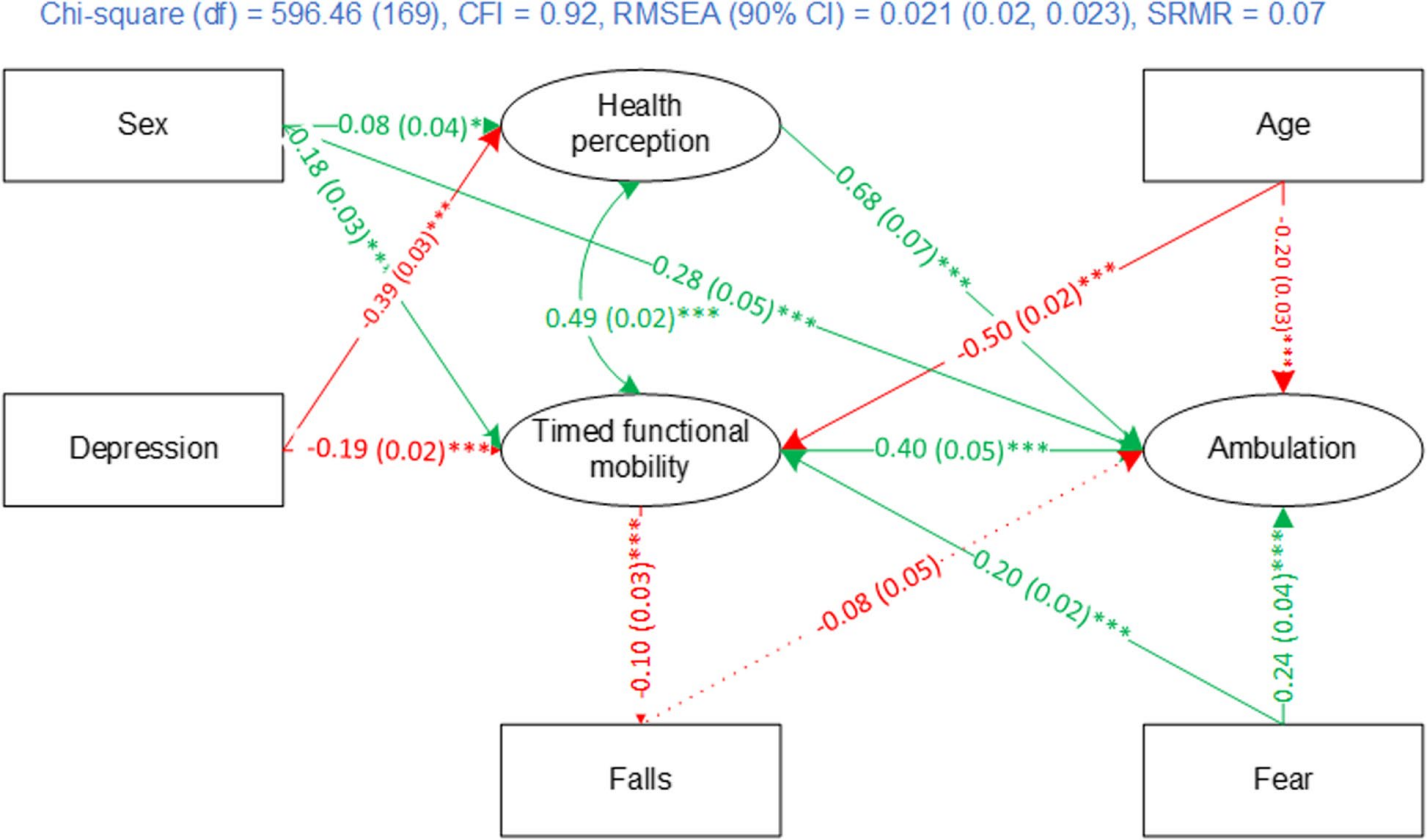
Structural model of the final community ambulation model for OA group. Note: green indicates positive association; red indicates negative association; cell format: path coefficient (standard error) ^significance level^ Path coefficients are not standardized. *** *p* < 0.001, * *p* < 0.05. Chi-square = robust chi-square test statistics, df = degree of freedom, CFI = robust comparative fit index, RMSEA = robust root mean square error of approximation, SRMR = standardized root mean residual

Then, age group, sex, and number of falls were added to the structural model. The SEM had close goodness-of-fit ((*χ*^2^ = 612.65, df = 168, CFI = 0.92, RMSEA (90% CI) = 0.022 (0.020–0.024), SRMR = 0.07). The path coefficient from age group to health perceptions was not significant; it was removed from the SEM. The modified model had similar fit ((*χ*^2^ = 596.46, df = 169, CFI = 0.92, RMSEA (90% CI) = 0.021 (0.020–0.023), SRMR = 0.07) to the data. Therefore, we selected this model as our final model. The measurement models and structural model in the final SEM for the OA group are shown in Figs. 5 and 6. Ambulation, health perceptions, and timed functional mobility represented better conditions.

Health perceptions (path coefficient = 0.68, *p* < .001), timed functional mobility (path coefficient = 0.40, *p* < .001), age group (path coefficient = − 0.20, *p* < .001), sex (path coefficient = 0.28, *p* < .001), and fear in walking alone after dark (path coefficient = 0.24, *p* < .001) were directly associated with ambulation. Depression was negatively associated with ambulation through its negative association with health perceptions (path coefficient = − 0.39, *p* < .001) and timed functional mobility (path coefficient = − 0.19, *p* < .001). Being male was associated with better ambulation (path coefficient = 0.28, *p* < .001), health perceptions (path coefficient = 0.08, *p* < .05) and timed functional mobility (path coefficient = 0.18, *p* < .001). Age group was negatively related to ambulation (path coefficient = − 0.20, *p* < .001) and timed functional mobility (path coefficient = − 0.50, *p* < .001).

Configural, metric and scalar invariance were supported across male and female groups and across age groups (younger than 65 and 65+) for the OA group. See Table 5. Therefore, the comparison of means of health perceptions, timed functional mobility, and community ambulation across sex and age groups are valid.

**Table 5:** Measurement invariance for sex (males vs. females) and age (65- vs. 65+) for the OA group

	Invariance type	*χ*^2^ (df)	∆ χ^2^ (∆df)	RMSEA	CFI	∆ CFI
Sex	Configural	891.58 (310)		0.026	0.970	
	Metric	861.68 (323)	−29.9 (13)	0.027	0.972	< 0.01
	Scalar	860.48 (350)	−1.2 (27)	0.023	0.973	< 0.01
Age	Configural	745.10 (310)		0.023	0.956	
	Metric	693.93 (323)	−51.17 (12)	0.020	0.962	< 0.01
	Scalar	726.20 (350)	32.27 (27)	0.020	0.962	< 0.01

***χ***^**2**^ *= robust* ***χ***^**2**^ *test statistics, df* degree of freedom, *CFI robust* comparative fit index*, RMSEA robust* root mean square error of approximation, **∆ =** *change.*

## Discussion

Using the CLSA baseline comprehensive cohort data, we developed community ambulation models for older adults aged 65+ and for people with OA aged 45 and older. A unique aspect of this study is that the models of community ambulation for people aged 65+ and those with OA used a large national dataset; using SEM allowed for the use of multiple indicator variables and covariates to be assessed together and identify the relationships between the variables and latent factors. The community ambulation post-stroke model was the basis for model development [25].

### Differences between original stroke model and older adult (65+) and OA models

The original model of community ambulation post stroke included three latent variables of ambulation (both indoor and outdoor), gait speed, and health perceptions. Though the outcome measures were different between that study and the current study, the constructs of ambulation and health perception are the same. Gait speed was expanded in the current study to include other timed tests known to be related to walking. The paths between gait speed/ timed functional mobility and ambulation; and between health perceptions and ambulation are in the same direction between models. Age and sex were not statistically significant covariates in the stroke model, but both were statistically significant in the older adult and OA models in relation to ambulation and timed functional mobility. Depression was a covariate that was significant in all models with higher depression leading to lower health perceptions in the stroke model and to lower health perceptions and lower timed functional mobility in the older adults and OA models. In the stroke model, walking endurance was proposed by people living with stroke as an important variable relating to community ambulation. In the 65+ and OA models, endurance was reflected as an indicator variable of the ambulation latent factor. The original stroke model did not include falls nor did it include fear of walking outdoors at night (as a reflection of the environment), which was suggested by people living with stroke as a component to consider. These aspects were both added as covariates to the current models.

### 65+ and OA models

As previously noted, 56% of the OA group was 65 or older, and 26.5% of the 65+ group had OA. Below, we discuss both models. It is essential to note that there was no statistical analysis carried out between the models for comparison, due to the overlap of participants. Each model stands alone for use in each population.

### 65+ model

#### Falls

Poorer timed functional mobility was associated with higher number of falls. This is supported in other literature in older adults, where low gait speed (below 1.0 m/sec) (in the timed functional mobility latent factor) is associated with numerous falls [54]. The number of falls in the previous 12 months, however, was not associated with the ambulation latent factor, despite most falls for older adults occurring while walking [55]. Participants in the CLSA were asked how many times they had fallen in the previous 12 months. It is possible that numbers of falls were not accurate due to recall bias. The reason for the falls identified were also not known.

#### Depression

In male older adults in the Longitudinal Aging Study Amsterdam, symptoms of depression were associated with lower gait speed [56]. Our findings showed an indirect association between depression and ambulation through timed functional mobility (of which gait speed is an indicator variable) for adults aged 65 + .

#### Health perception

Health perception was positively associated with ambulation; this is supported in the older adult literature. General health perception is a variable in the latent factor of health perception. In a Canadian study, older adults recently discharged from hospital with poor self-rated general health were 3.9 times more likely to have difficulty walking in the community compared to those with good self-rated general health [57]. A study in Japan found that there was a trend of a higher proportion of people with fair or poor self-rated health when the frequency of going outdoors was low [3].

#### Timed functional mobility

The latent factor of timed functional mobility (including variables of gait speed, sit to stand / leg strength, TUG and balance) was positively associated with the ambulation latent factor. The positive association between higher gait speed and higher ambulation in the community is supported in the literature. Self-selected gait speed is a predictor of meeting suggested walking guidelines for older adults, measured in steps-per-day [58]. This current study used comfortable (self-selected) gait speed in the model. In a previous study, older adults who walked more (measured by ≥7000 steps per day) were more likely to have better leg strength, using a STS test [59]. Our results agree with that finding; we found that a shorter time to complete 5 STS means higher leg strength, as part of timed functional mobility, which was positively associated with ambulation.

#### Fear/safety

Most older adults living in an urban setting in England did not feel safe walking outside at night for a number of reasons, including fear of crime [60]. In this current study a positive association between lower fear in walking alone at night and better timed functional mobility and ambulation was identified.

#### Age

For those 65+, being in an older age group (75+) was associated with lower health perceptions, lower ambulation and lower timed functional mobility. The health perceptions latent factor included self-reported general health. A study of 8905 Americans, however, found that adults aged 75–84 and 85+ reported better self-rated health compared to those aged 65–74 [61]. Interestingly, in the same study, data from 4442 older adults from China showed no association between age and self-reported health [61]. It has been shown that norms for gait speed (as an indicator of timed functional mobility) are slower in older age groups relative to younger age groups [62].

#### Sex

In this study, being male was positively associated with higher health perceptions, ambulation and timed functional mobility in adults 65+. A meta-analysis of gait speed norms demonstrated that men have higher gait speeds than women, by age group [62]. Older women have been noted to participate less in walking and other physical activity than men [63].

### OA model

#### Falls

In a systematic review, factors associated with falling in OA included decreased balance, decreased strength and pain [64]. In this study, decreased balance and decreased strength are represented in the timed functional mobility latent factor, through a negative association with falls, suggesting that lower timed functional mobility (e.g. lower strength and lower balance) is associated with a higher number of falls. Pain is represented in the health perceptions latent factor, which is associated with number of falls indirectly through a covariance with timed functional mobility. Like the older adult model, the path from the number of falls to ambulation was negative, but not statistically significant.

#### Depression

Higher levels of depression were associated with lower performance of timed functional mobility tasks in the OA model. Gait speed is one of the variables in the timed functional mobility latent factor. Slow gait speed, often used as a proxy for challenges with community ambulation, has been shown to be associated with higher levels of depression and worsening depression in people with OA of the knee [65].

#### Health perception

Pain is a component of the health perception latent factor. A systematic review of pain and physical functioning in knee OA found that higher knee pain was associated with a decline of physical functioning; walking is considered to be an aspect of physical functioning [66]. Less pain (as a variable in health perceptions latent factor) was positively associated with ambulation. In our modeling, a higher score represents lower pain and less activities prevented by pain, meaning that less pain is associated with more ambulation, supporting the systematic review findings above.

#### Timed functional mobility

We found that higher levels of timed functional mobility (gait speed, leg strength, and balance) were associated with higher levels of ambulation for those with OA. An observational study of over 3000 individuals with knee OA found that if not walking was replaced by walking for 5–20 minutes at a moderate to vigorous intensity, there was a statistically significant decrease in the risk of having a gait speed of less than 1.0 m/s [67]. Gait speeds of 0.8 m/second or greater are suggestive of being a community ambulator, for any adult population and slower gait speeds are associated with increased risk of falls and being more likely to be hospitalized [68].

#### Fear / safety

In this current study, the model for those with OA showed an association between the finding of lower fear in walking alone at night and better timed functional mobility and ambulation. This is supported by a previous study showing that perception of neighbourhood safety was positively associated with outdoor walking for people with OA [69].

#### Age

It has been noted that for people with OA, difficulty in walking more than one mile increases over the age of 65 compared to those in younger age groups [16]. This is similar to our finding that for people with OA, older age was associated with lower ambulation and lower timed functional mobility.

#### Sex

Similar to the 65+ group, being male was associated with higher health perceptions, ambulation and timed functional mobility in people with OA. This finding is supported in the literature. Compared to men, women with OA report greater difficulty with activities such as climbing up and down stairs and walking 500 m without walking aids [70], and as age increases, women with arthritis report higher walking limitations than men [16]. Additionally, using the Tracking cohort of the CLSA (distinct from the cohort in the current study), it was also found that Canadian adults with OA of the lower extremity, aged 45 and over, were less likely to walk outside their home and yard if they were female [24].

### Clinical implications

Rehabilitation professionals regularly assess aspects of timed functional mobility such as balance, leg strength and gait speed when a client has goals related to community ambulation. The models include these important clinical components. As a variable related to health perception, the level of pain that a client experiences and how it interferes with function are often discussed, however, rehabilitation professionals may also consider the evaluation of self-rated health (general health) with their clients, which could lead to further discussion during history-taking with clients regarding challenges and difficulties in timed functional mobility and ambulation. Another area addressed by the model is the covariate of fear in walking in the community after dark, which is one aspect of the physical environment in which a person lives that could affect community ambulation [21]. It is an important reminder to consider evaluating an individual’s environment when developing community ambulation programs or addressing community ambulation goals. The finding of depression being related to lower timed functional mobility is of importance. Rehabilitation professionals may incorporate screening for depression in their client assessments and consult with other health care professional team members to address treatment of depression in their clients where appropriate.

As noted above, we found that in both the model for people with OA (45+) and for older adults (65+), males were associated with higher levels of health perception, timed functional mobility and ambulation. However, the measurement invariance analysis showed that the means of the latent factors can be interpreted the same way for males and females. It is useful for clinicians and researchers to know that the same latent factors of health perception, timed functional mobility and community ambulation expressed in the models can be applied to both males and females when choosing assessment and intervention strategies.

### Missing data

To handle the missing data, pairwise deletion was used. Pairwise deletion and listwise deletion are the only two options to handle missing data when using the WLSMV estimator in lavaan [42]. Pairwise deletion uses all cases that have data present for each variable or each pair of variables, which will retain more information than listwise deletion [71]. Moreover, listwise deletion can result in substantially reduced sample size and lower statistical power, especially when having large number of variables such as the case in the current study [71]. Pairwise deletion method in SEM has shown to produce unbiased parameter estimates and standard errors when a reasonable sample size is used [72, 73]. Therefore, we choose to use the pairwise deletion instead of listwise deletion to handle missing data. However, there are a few disadvantages of the pairwise deletion method [71]. First, the correlation matrix may be not positive definite, so that parameter estimating is impossible. However, the rate of having this problem is very low even when the percent of missing is large (i.e., 50%) and the sample size is small (i.e., 100) [72]. We did not have this parameter estimating issue when fitting the model. Another concern is the chi-square test statistics can be greatly biased [72]. However, in the current study, we did not rely on chi-square test statistics for goodness-of-fit and model comparison as they are also very sensitive to sample size. Therefore, our conclusions on the final models should not be affected by this disadvantage. An important assumption of using pairwise deletion is missing completely at random. It is difficult to argue if this assumption is met in any study. Multiple imputation, which is a recommended method to handle missing, can be explored in future studies. Multiple imputation also requires the missing at random assumption. Therefore, in the current study, we used pairwise deletion because the percent of missing was less than 1% for most variables in our model, however, two observed variables (i.e., balance and standing up – leg strength) had 10.3% and 8.6% missing in the OA group, likely due to contraindications to testing.

### Limitations

Since this was a secondary data analysis, we were limited to the variables available in the CLSA dataset. Many of the variables used were from different outcome measures than the original model for individuals post-stroke, but the overall constructs were consistent. We were not able to use the CLSA sample weights, since the “lavaan” package with WLSMV estimation does not allow sample weights.

We focused on two groups in this study – older adults aged 65–85 and those with OA aged 45–85. There is overlap of individuals between the model of those with OA and people 65+; 56% of the OA group was 65 or older. For this reason, we did not compare the models statistically; they are discrete models, developed for use in different scenarios. Self-declared gender identity was not available in the baseline comprehensive dataset which we used, however, it is available for follow-up evaluations, and should be included in future analyses. It was not known if individuals with OA had multiple hip and/or knee joints affected; this could potentially affect the amount of community ambulation that an individual would participate in. Future studies should investigate this further.

The data was from a large Canadian database. The Comprehensive cohort is national, but not considered nationally representative; participants live in locations that are 25–50 km from city Data Collection Sites to allow for in-person testing [74]. The models are not predictive of future community ambulation, but demonstrate an association between variables and latent factors. Future research could include using the models with CLSA follow-up data to investigate how health perceptions and timed functional mobility are associated with ambulation at a later point in time.

## Conclusions and implications

Many associations in the SEMs are supported by previous studies which evaluated the relationships of numerous variables to community ambulation. This study combined self-report and observed measures of physical function to describe models of community ambulation for adults age 65+ and for people with OA of the hip and / or knee aged 45+, based on a previously developed model post-stroke.

Many of the indicators of the three latent factors (ambulation, timed functional mobility, and health perceptions) are specifically addressed in rehabilitation-based programs and community-based exercise programs that focus on identified limitations and challenges in community ambulation. For example, the indicators of walking ability, walking outdoors, walking endurance, pain, gait speed, leg strength, balance and functional speed are often addressed in rehabilitation and community based programs, as determined by the goals of the clients / participants. As noted in the discussion, additional consideration of self-rated health, depression and the physical environment in which the client lives should be considered. Community prevention programs could also address these variables with the aim of preventing mobility decline and decreasing mortality risk.

Community organizations across Canada, which focus on promoting active aging and preventing the effects of inactivity often provide peer-led exercise and walking programs; such programs could also utilize results of this project in identifying components which could be included in programming.

Researchers can use the models of community ambulation to frame and conceptualize research on community ambulation, with the focus on contributing variables, outcomes, or interventions. Using theoretical frameworks such as this model of community ambulation can help to advance the development and evaluation of community ambulation interventions.

### Supplementary Information

**Additional file 1.**

**Additional file 2.**

**Additional file 3.**

**Additional file 4.**

## Data Availability

Data are available from the Canadian Longitudinal Study on Aging (www.clsa-elcv.ca) for researchers who meet the criteria for access to de-identified CLSA data.

## Abbreviations

*χ*^2^: Chi-square
CES-D 10: Center for Epidemiologic Studies Short Depression Scale
CFI: Comparative fit index
CI: Confidence interval
CLSA: Canadian Longitudinal Study on Aging
df: Degrees of freedom
HRS: Health and Retirement survey
LSI: Life Space Index
OA: Osteoarthritis
OARS: Older Americans Resources and Services
PASE: Physical Activity Scale for the Elderly
RMSEA: Root mean square error of approximation
SEM: Structural equation modeling
TUG: Timed up and go
WLSMV: Weighted least square mean and variance
