# Supplementary material for: Community ambulation in older adults and people with OA – a model verification using Canadian Longitudinal Study on Aging (CLSA) data
Source: BMC Geriatr. 2024 Jan 6;24:31. doi: 10.1186/s12877-023-04598-3 (PMC10771682; doi:10.1186/s12877-023-04598-3)
Supplement: Supplementary file 3 — Additional file 3. [file 12877_2023_4598_MOESM3_ESM.docx]

**Table S3 Goodness-of-fit of equivalent models for 65+ cohort**

|  | Community ambulation | | | | | |
| --- | --- | --- | --- | --- | --- | --- |
|  |  | Model | \| chisq(df) \| \| --- \| | CFI | RMSEA (90%CI) |  |
|  | Final model in manuscipt | ambulation =~ LSI_NGHBAID_COM_NEW + LSI_NGHBFQ_COM_NEW + LSI_OUTAID_COM_NEW + LSI_OUTFQ_COM_NEW + LSI_RMAID_COM_NEW + LSI_RMFQ_COM_NEW + LSI_TWNAID_COM_NEW + LSI_TWNFQ_COM_NEW + ADL_CR2_WALK_COMBINED + PA2_WALK_MCQ + PA2_WALK_WALKHR health_perception =~ GEN_HLTH_COM + HUP_FREE_PRVACT_COMBINE + HUP_PAIN_INTENSITY_COMBINED timed_functional_mobility = ~ TUG_TIME_COM + NUM_WALK_GAIT_scaled + CR_TIME_COM + BAL_BEST_COM_Scaled ambulation ~ health_perception + timed_functional_mobility ambulation ~ AGE_GRP_COM + FALLS_COMBINED_MCQ + SEX_ASK_COM + ENV_AFRDWLK_MCQ  health_perception ~ DEP_FLDP_COM + AGE_GRP_COM + SEX_ASK_COM timed_functional_mobility ~ AGE_GRP_COM + DEP_FLDP_COM + SEX_ASK_COM + ENV_AFRDWLK_MCQ  FALLS_COMBINED_MCQ ~ gait_speed | 983.46 (208) *** | 0.91 | 0.018 (0.017, 0.019) |  |
| equivalent models | fall as covariate for timed functional mobility | ambulation =~ LSI_NGHBAID_COM_NEW + LSI_NGHBFQ_COM_NEW + LSI_OUTAID_COM_NEW + LSI_OUTFQ_COM_NEW + LSI_RMAID_COM_NEW + LSI_RMFQ_COM_NEW + LSI_TWNAID_COM_NEW + LSI_TWNFQ_COM_NEW + ADL_CR2_WALK_COMBINED + PA2_WALK_MCQ + PA2_WALK_WALKHR health_perception =~ GEN_HLTH_COM + HUP_FREE_PRVACT_COMBINE + HUP_PAIN_INTENSITY_COMBINED timed_functional_mobility = ~ TUG_TIME_COM + NUM_WALK_GAIT_scaled + CR_TIME_COM + BAL_BEST_COM_Scaled ambulation ~ health_perception + timed_functional_mobility ambulation ~ AGE_GRP_COM + FALLS_COMBINED_MCQ + SEX_ASK_COM + ENV_AFRDWLK_MCQ  health_perception ~ DEP_FLDP_COM + AGE_GRP_COM + SEX_ASK_COM timed_functional_mobility ~ AGE_GRP_COM + DEP_FLDP_COM + SEX_ASK_COM + ENV_AFRDWLK_MCQ + FALLS_COMBINED_MCQ | 1074.22 (204) *** | 0.86 | 0.019 (0.018, 0.020) |  |
|  | removed path between timed functional mobility and falls | ambulation =~ LSI_NGHBAID_COM_NEW + LSI_NGHBFQ_COM_NEW + LSI_OUTAID_COM_NEW + LSI_OUTFQ_COM_NEW + LSI_RMAID_COM_NEW + LSI_RMFQ_COM_NEW + LSI_TWNAID_COM_NEW + LSI_TWNFQ_COM_NEW + ADL_CR2_WALK_COMBINED + PA2_WALK_MCQ + PA2_WALK_WALKHR health_perception =~ GEN_HLTH_COM + HUP_FREE_PRVACT_COMBINE + HUP_PAIN_INTENSITY_COMBINED timed_functional_mobility = ~ TUG_TIME_COM + NUM_WALK_GAIT_scaled + CR_TIME_COM + BAL_BEST_COM_Scaled ambulation ~ health_perception + timed_functional_mobility ambulation ~ AGE_GRP_COM + FALLS_COMBINED_MCQ + SEX_ASK_COM + ENV_AFRDWLK_MCQ  health_perception ~ DEP_FLDP_COM + AGE_GRP_COM + SEX_ASK_COM timed_functional_mobility ~ AGE_GRP_COM + DEP_FLDP_COM + SEX_ASK_COM + ENV_AFRDWLK_MCQ | 1782.69 (205) *** | 0.74 | 0.026 (0.025, 0.027) |  |
