# Supplementary material for: Community ambulation in older adults and people with OA – a model verification using Canadian Longitudinal Study on Aging (CLSA) data
Source: BMC Geriatr. 2024 Jan 6;24:31. doi: 10.1186/s12877-023-04598-3 (PMC10771682; doi:10.1186/s12877-023-04598-3)
Supplement: Supplementary file 4 — Additional file 4. [file 12877_2023_4598_MOESM4_ESM.docx]

Figure S1 Alternative 65+ model: Path from timed functional mobility to fear


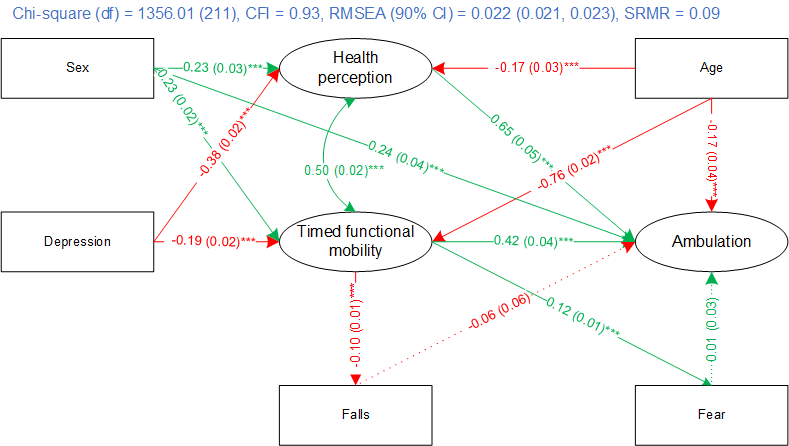


*Note: green indicates positive association; red indicates negative association; cell format: path coefficient (standard error) ­­^significance level^ Path coefficients are not standardized.*

**** p<0.001*

*Chi-square = robust chi-square test statistics, df = degree of freedom, CFI= robust* comparative fit index*, RMSEA = robust* root mean square error of approximation, SRMR = standardized root mean residual
